# Blood-Brain Barrier Disruption Increases Amyloid-Related Pathology in TgSwDI Mice

**DOI:** 10.3390/ijms22031231

**Published:** 2021-01-27

**Authors:** Ihab M. Abdallah, Kamal M. Al-Shami, Euitaek Yang, Amal Kaddoumi

**Affiliations:** Department of Drug Discovery and Development, Harrison School of Pharmacy, Auburn University, 720 S Donahue Drive, Auburn, AL 36849, USA; iza0012@auburn.edu (I.M.A.); kma0058@auburn.edu (K.M.A.-S.); ezy0014@auburn.edu (E.Y.)

**Keywords:** Alzheimer’s disease, CAA, blood-brain barrier, amyloid-β, P-glycoprotein, breast cancer resistance protein

## Abstract

In Alzheimer’s disease (AD), several studies have reported blood-brain barrier (BBB) breakdown with compromised function. P-glycoprotein (P-gp) and breast cancer resistance protein (BCRP) are transport proteins localized at the BBB luminal membrane and play an important role in the clearance of amyloid-β (Aβ). The purpose of this study was to investigate the effect of pharmacological inhibition of Aβ efflux transporters on BBB function and Aβ accumulation and related pathology. Recently, we have developed an in vitro high-throughput screening assay to screen for compounds that modulate the integrity of a cell-based BBB model, which identified elacridar as a disruptor of the monolayer integrity. Elacridar, an investigational compound known for its P-gp and BCRP inhibitory effect and widely used in cancer research. Therefore, it was used as a model compound for further evaluation in a mouse model of AD, namely TgSwDI. TgSwDI mouse is also used as a model for cerebral amyloid angiopathy (CAA). Results showed that P-gp and BCRP inhibition by elacridar disrupted the BBB integrity as measured by increased IgG extravasation and reduced expression of tight junction proteins, increased amyloid deposition due to P-gp, and BCRP downregulation and receptor for advanced glycation end products (RAGE) upregulation, increased CAA and astrogliosis. Further studies revealed the effect was mediated by activation of NF-κB pathway. In conclusion, results suggest that BBB disruption by inhibiting P-gp and BCRP exacerbates AD pathology in a mouse model of AD, and indicate that therapeutic drugs that inhibit P-gp and BCRP could increase the risk for AD.

## 1. Introduction

Alzheimer’s disease (AD) is a progressive neurodegenerative disorder and the most common cause of dementia [1,2]. It is considered the sixth leading cause of death among Americans above 65 years old. The pathogenesis of AD is complex, and to date no effective treatment exists. AD is characterized by the deposition and accumulation of amyloid-β (Aβ) in the brain parenchyma and cerebral vessels [3,4]. Aβ accumulation in the brain parenchyma forms senile plaques, whereas the extracellular deposition of Aβ in cerebral vessels forms cerebral amyloid angiopathy (CAA) [5,6]. Aβ peptides are derived from the proteolytic cleavage of the amyloid precursor protein (APP) to form Aβ_40_ and Aβ_42_. Aβ generated in the brain can be eliminated through transport proteins across the blood-brain barrier (BBB), enzymatic degradation, and perivascular drainage via the vascular basement membrane [7].

Dysfunction of the BBB could be involved in the pathogenesis of AD and CAA [5]. While additional studies are necessary to clarify the mechanism of BBB dysfunction, available reports support that disruption of Aβ clearance across the BBB may result in exacerbated Aβ accumulation in the brain [8,9,10]. BBB integrity is strictly controlled by cells and basement membranes in the neurovascular unit in physiological conditions. However, the barrier function is likely compromised during aging and AD [11].

The BBB is composed of a basement membrane, astrocytes, pericytes, and endothelial cells. The endothelial cells are tightly connected at a junctional complex via tight junction and adherence junction proteins [12], which limit the transport of solutes from the blood to the brain and vice versa [13]. Aβ transport across the BBB is bidirectional where its transport from the brain to blood and vice versa requires carrier- or receptor-mediated transport proteins [14]. Efflux transporters P-glycoprotein (P-gp, ABCB1), breast cancer resistant protein (BCRP, ABCG2), and low-density lipoprotein receptor-related protein-1 (LRP-1) regulate the traffic across the BBB to the blood and play key roles in brain Aβ homeostasis [14,15]. In comparison, the receptor for advanced glycated end products (RAGE) in the endothelial cells is able to transport Aβ from blood to brain across the BBB [16].

P-gp and BCRP belong to the family of ATP-binding cassette transporters and are highly expressed at the luminal side of BBB endothelium [17]. Both proteins function as efflux transporters to limit foreign molecules access to the brain and extrude substances from brain to blood to protect the brain from potentially toxic substances [18]. In normal aging and AD, P-gp, and BCRP are downregulated [19]. In addition, several lines of evidence have shown that compromised BBB integrity and function are associated with altered protein expression and cellular secretions, and inflammatory activation [20]. Studies reported that reduced expression/function of P-gp and BCRP led to a reduction in the clearance of Aβ and increase in Aβ-related pathology [21,22,23,24], while restoring the function of P-gp at the BBB increased Aβ clearance from the brain [25]. These findings suggest that P-gp and BCRP play an important role in BBB function and suggest that an alteration in these transporters’ functions could alter the BBB function and brain homeostasis.

Aging is associated with an increased risk of chronic diseases necessitating the prescription of multiple medications, which could independently and/or through drug-drug interaction inhibit transporters function and thus affect the BBB. In this work, we sought to study the effect of pharmacological disruption of BBB via P-gp and BCRP inhibition on Aβ pathology in TgSwDI, a mouse model for AD and CAA. For this, the third generation P-gp and BCRP inhibitor elacridar [26] was used as a model compound to investigate the effect of both transporters inhibition on BBB function and on Aβ levels in the brains of TgSwDI mice. Elacridar was identified as a BBB disruptor by our recently developed high throughput screening (HTS) assay using a cell-based BBB model [27].

## 2. Results

### 2.1. P-gp and BCRP Inhibition by Elacridar Reduced the Cell-Based BBB Model Function through Activation of NF-κB Pathway in bEnd3 Cells

**Figure 1:**
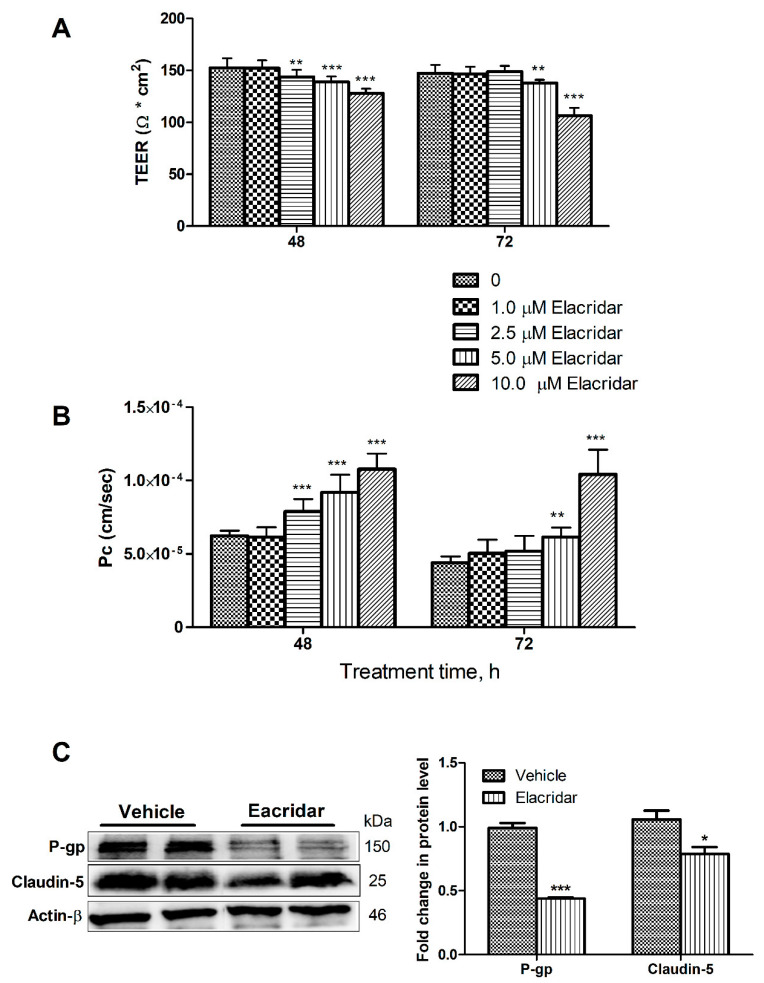
Effects of elacridar on the barrier function of bEnd3 cells. (**A**) Exposing bEnd3 cells monolayers to elacridar resulted in the reduction of TEER measurements. TEER unit is in Ω * cm^2^. (**B**) Elacridar significantly increased the permeability (Pc, cm/sec) of LY, a permeation marker, in a concentration and time-dependent manner. (**C**) Representative Western blot and densitometry analysis of P-gp and claudin-5 in bEnd3 cells, presented as fold change by elacridar compared to vehicle treatment, showed elacridar significantly reduced both proteins expression when compared to vehicle treated group. Statistical analysis was determined by one-way ANOVA test for (**A**,**B**) and Student’s *t*-test for (**C**). Data are presented as mean ± SD of at least three independent experiments. * *p* ≤ 0.05, ** *p* ≤ 0.01, and *** *p* ≤ 0.001. kDa indicates the molecular weight of analyzed proteins.

To confirm our previous HTS findings for elacridar’s disruptive effect on the integrity of a cell-based BBB model, in vitro concentration-dependent studies were performed using bEnd3 cells. The effect of increasing concentrations of elacridar on the barrier function of the monolayer is shown in Figure 1. Elacridar treatment for 48 and 72 h resulted in a concentration-dependent reduction in transendothelial electrical resistance (TEER) measurements where the monolayer tightness was disrupted by elacridar in the concentration range from 2.5–10 µM with treatment times of 48 and 72 h when compared to vehicle-treated cells (Figure 1A). The reduction in TEER values was associated with a significant increase in the permeability of Lucifer yellow (LY) across the monolayer (Figure 1B), suggesting a leaky monolayer that is consistent with results observed with TEER measurements. Besides, results from Western blotting showed the inhibitory effect of elacridar on P-gp and claudin-5 expressions (Figure 1C).

**Figure 2:**
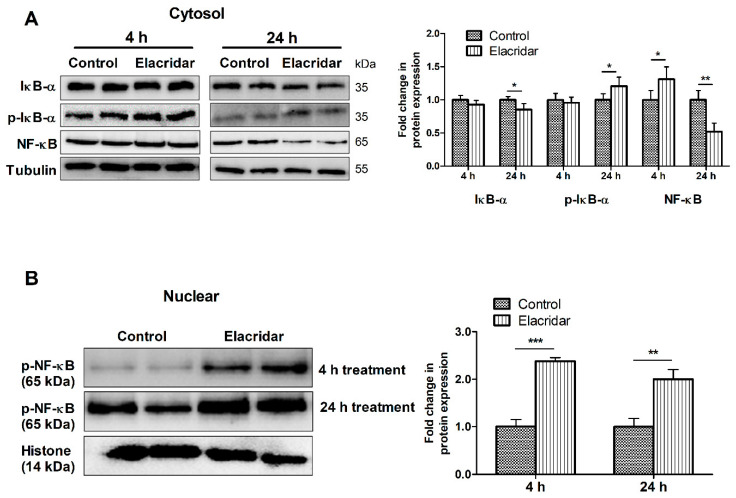
Treatment with elacridar (5 µM) significantly activated the NF-κB pathway in cultured bEnd3 cells in vitro. (**A**) Representative Western blot and densitometry analysis of the cytosolic fraction of IκB-α, p-IκB-α, and NF-κB, presented as fold change by elacridar compared to vehicle treatment, at 4 and 24 h post-treatment. (**B**) Representative Western blot and densitometry analysis of the nuclear fraction of p-NF-κB, presented as fold change by elacridar compared to vehicle treatment, at 4 and 24 h post-treatment. Statistical analysis was determined by Student’s *t*-test. Data are presented as mean ± SD of 3 independent experiments. * *p* < 0.05, ** *p* < 0.01, *** *p* < 0.001 compared to control. kDa indicates the molecular weight of analyzed proteins.

To study the role of NF-κB signaling in the observed outcome of the leaky monolayer, bEnd3 cells were treated with 5 µM elacridar for 4 and 24 h and then analyzed by Western blot for NF-κB signaling pathway in cytosolic and nuclear fractions. As shown in Figure 2A, in the cytosolic fraction, elacridar significantly reduced the expression of inhibitor of NF-κB (IκB-α) at 24 h post-treatment. This reduction in IκB-α was paralleled with a significant increase of cytosolic p-IκB-α as an indicator of activated degradation of IκB-α in response to elacridar. Elacridar treatment for 4 h did not demonstrate any changes in IκB-α and its phosphorylated form. However, elacridar’s effect on NF-κB was different, where at 4 h treatment, NF-κB levels were higher than control, which was reversed following 24 h treatment (Figure 2A). Furthermore, elacridar increased the translocation of p-NF-κB in the nuclear fraction following 4 and 24 h of treatment, as shown in Figure 2B.

### 2.2. Elacridar Disrupted the BBB Integrity in TgSwDI Mice

**Figure 3:**
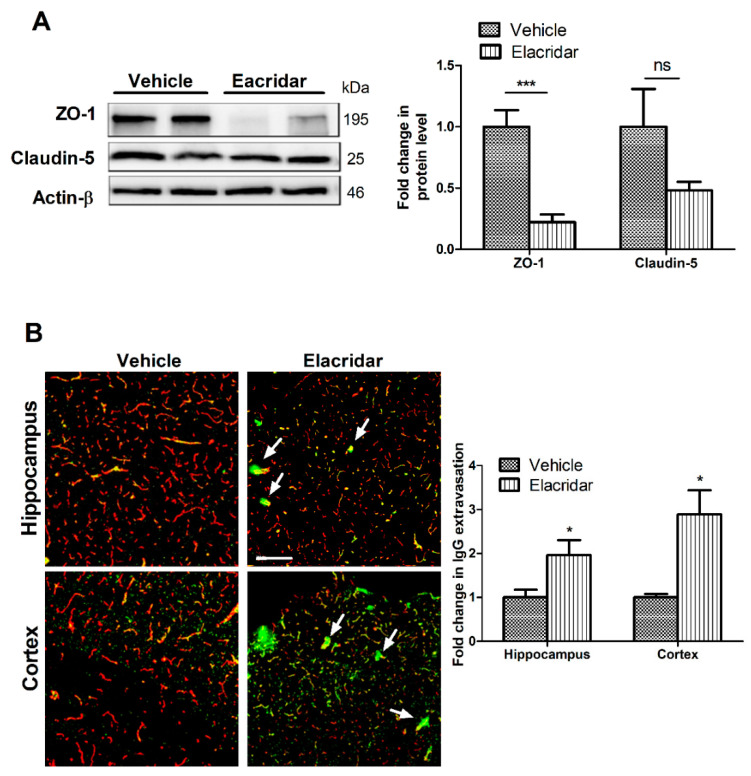
Treatment with elacridar (10 mg/kg) i.p. for 28 days disrupted BBB integrity in TgSwDI mice. (**A**) Representative Western blot and densitometry analysis of ZO-1 and claudin-5 in vivo from microvessels isolated from mouse brains. Elacridar treatment significantly decreased the expression of ZO1 in isolated microvessels from mouse brains when compared to the vehicle-treated group. kDa indicates the molecular weight of analyzed proteins. (**B**) Representative brain sections stained with anti-mouse IgG antibody to detect IgG extravasation (green) and anti-collagen antibody (red), with their optical density quantification in mouse brain hippocampus and cortex. White arrows indicate BBB leakage as demonstrated by IgG extravasation (green). Elacridar increased IgG extravasation in mouse hippocampus and cortex compared to vehicle-treated mice. Scale bar = 100 µm. Statistical analysis was determined by Student’s *t*-test. Data represented as mean ± SEM of *n* = 5 mice per group. ns = not significant; * *p* < 0.05, *** *p* < 0.001 compared to vehicle.

To investigate the effect of TgSwDI mice treatment with elacridar for 28 days on the BBB integrity, the expression of tight junction proteins ZO-1 and claudin-5 in the isolated microvessels from mouse brains were analyzed by Western blot, and levels of IgG extravasation in mouse brains by immunostaining. Elacridar treatment significantly reduced the expression of ZO-1 by >75% when compared to vehicle-treated mice. In addition, claudin-5, while it did not reach a statistically significant level, it showed about 50% reduction in its expression (Figure 3A). This effect was associated with a significant increase in IgG extravasation in the cortexes and hippocampi of mouse brains (Figure 3B), where IgG leakiness was obvious surrounding the vessels (white arrows). These results suggest that elacridar disrupted the BBB intactness in TgSwDI mouse brains.

### 2.3. Elacridar Reduced the Expression of Aβ Transport Proteins in Isolated Microvessels from TgSwDI Mouse Brains

**Figure 4:**
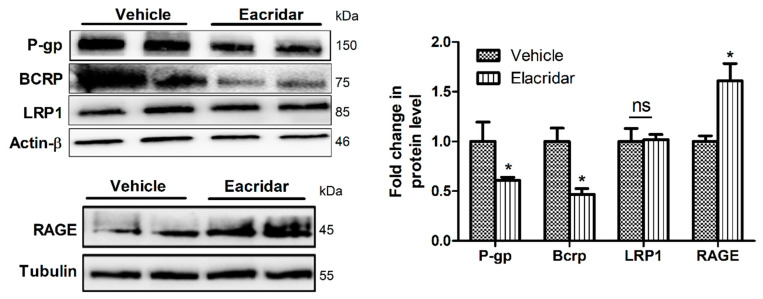
Treatment with elacridar (10 mg/kg) i.p. for 28 days altered the expression Aβ transport proteins in isolated microvessels from TgSwDI mouse brains. Representative Western blot and densitometry analysis of P-gp, BCRP, LRP1, and RAGE, presented as fold change by elacridar on each protein compared to vehicle treatment, demonstrated that elacridar reduced the expression of P-gp and BCRP, while increased RAGE. Statistical analysis was determined by Student’s *t*-test. Data represented as mean ± SEM of *n* = 5 mice per group, ns = not significant; * *p* < 0.05 compared to vehicle-treated mice. kDa indicates the molecular weight of analyzed proteins.

Figure 4 demonstrates the effect of elacridar on Aβ efflux transporters in isolated microvessels from mouse brains as measured by Western blotting. As expected, elacridar significantly downregulated the expression of P-gp by 40% and BCRP by 53% without altering LRP1 levels. On the other hand, elacridar significantly induced RAGE expression by approximately 60% (Figure 4).

### 2.4. BBB Disruption by Elacridar Increased Aβ Load in TgSwDI Mouse Brains

**Figure 5:**
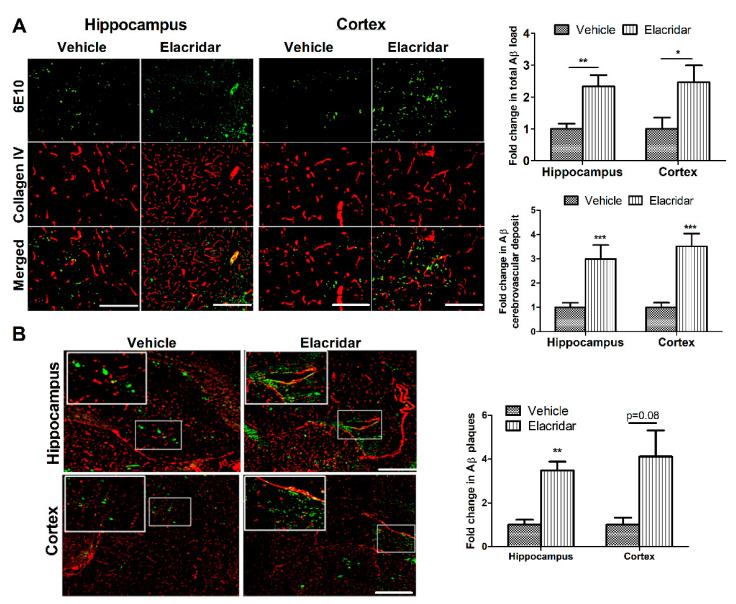
Elacridar treatment increased brain Aβ burden in the brains of TgSwDI mice. (**A**) Representative brain sections from mice cortex and hippocampus regions stained with 6E10 (green) antibody against Aβ to detect total Aβ load and anti-collagen IV (red) to stain microvessels. Semi-quantification analysis of both regions showed a significant increase in parenchymal Aβ burden and cerebrovascular Aβ deposit. (**B**) Representative brain sections stained with ThioS (green) and anti-collagen IV (red) to stain microvessels in cortex and hippocampus regions, with the corresponding quantification of the area covered with Aβ plaques (ThioS). The top white square is a magnification of the small square showing increased Aβ deposit on the microvessels caused by elacridar. The semi-quantification analysis is presented as fold change caused by elacridar when compared to vehicle treatment. Scale bar = 100 μm. Statistical analysis was determined by Student’s *t*-test. Data are presented as mean ± SEM of *n* = 5 mice per group, * *p* < 0.05, ** *p* < 0.01, *** *p* < 0.001 compared to vehicle-treated group.

Compared to vehicle-treated mice, elacridar significantly increased total Aβ load in mouse brains as determined by immunohistochemical analysis (Figure 5). Aβ deposition on microvessels, besides parenchyma, was also observed as detected by its co-localization with collagen-IV used as a marker for microvessels. Semi-quantitative analysis of parenchymal and cerebrovascular Aβ deposits showed elacridar caused a significant increase by more than 2.3- and 3-fold in the cortexes and hippocampi of mouse brains, respectively, when compared to the vehicle-treated group (Figure 5A). In addition, elacridar treatment increased Aβ plaques deposition in the brain parenchyma and microvessels as determined by Thioflavin S (ThioS) staining (Figure 5B). These results suggest elacridar further induced CAA, an effect that was associated with the downregulation of Aβ clearance proteins P-gp and BCRP, and upregulation of RAGE.

### 2.5. BBB Disruption by Elacridar Increased Astrocytes Activation and Matrix Metallopeptidase 9 (MMP9) Levels

**Figure 6:**
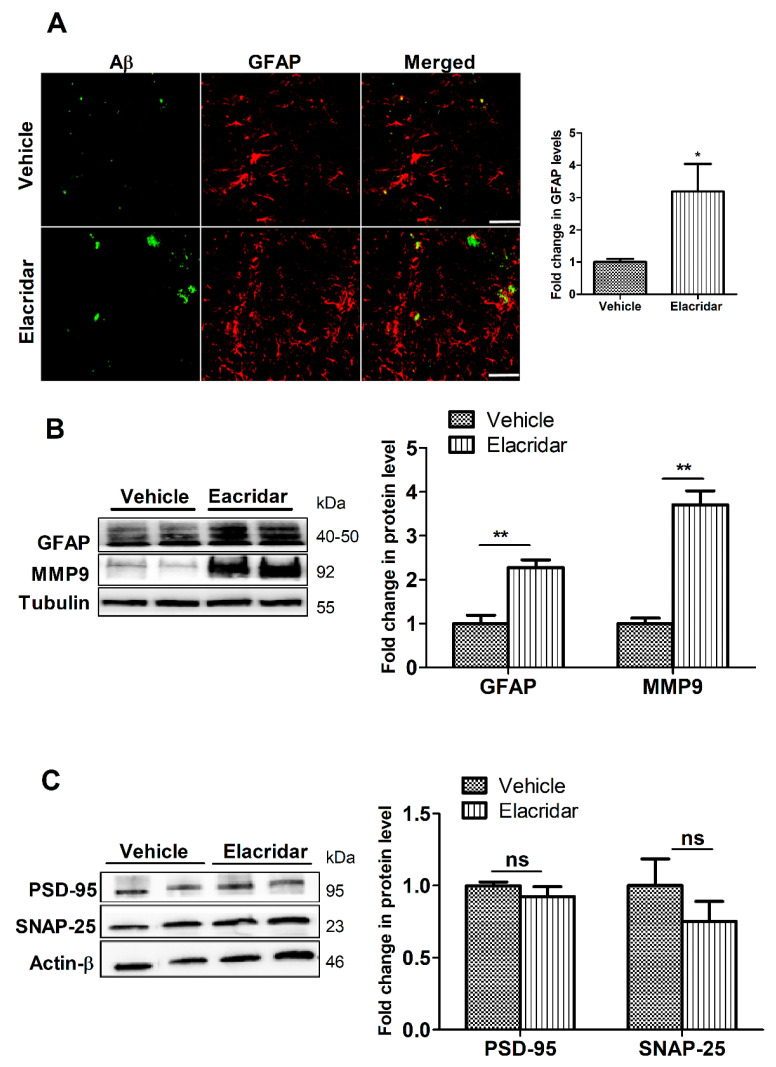
Elacridar treatment (10 mg/kg/day i.p. for 28 days) significantly increased astrogliosis marker GFAP in TgSwDI mouse brains. (**A**) Representative brain sections from mouse hippocampus stained with GFAP antibody (red) to stain activated astrocytes and with 6E10 (green) antibody to detect total Aβ, with the corresponding quantification of GFAP. Scale bar, 50 μm. The semi-quantification analysis is presented as fold change caused by elacridar when compared to vehicle treatment. (**B**) Representative Western blot and densitometry analysis of GFAP expressions in mouse brain homogenates. (**C**) Representative Western blot and densitometry analysis of PSD-95 and SNAP-25 expressions in mouse brain homogenates. Data from Western blot is presented as fold change by elacridar on each protein compared to vehicle treatment. Statistical analysis was determined by Student’s *t*-test. Data are presented as mean ± SEM for *n* = 5 mice per group. ns = not significant, * *p* < 0.05, ** *p* < 0.01, compared to vehicle-treated group. kDa indicates the molecular weight of analyzed proteins.

To evaluate the effect of BBB disruption by elacridar on astrogliosis, a feature of Aβ-related pathology, we assessed astrocytes activation and glial fibrillary acidic protein (GFAP) levels by immunostaining and Western blot. Results demonstrated a significant increase in the number of GFAP-positive astrocytes that was associated with an increase in cells branching in the hippocampal region of mouse brains (Figure 6A). Consistent with these results, findings from Western blot demonstrated elacridar significantly increased GFAP levels by 128% compared to vehicle-treated mice (Figure 6B). In addition, elacridar significantly increased MMP9 levels in mouse brains by 3.7-fold (Figure 6B).

### 2.6. Elacridar Treatment Did Not Alter the Expression of Synaptic Markers

Two synaptic markers were evaluated, the pre-synaptic marker synaptosomal-associated protein-25 (SNAP-25) and the postsynaptic marker postsynaptic density protein-95 (PSD-95). As shown in Figure 6C, mice treated with elacridar, while demonstrated a trend for reduction, the effect on altering the expression of PSD-95 or SNAP-25 was not significant.

### 2.7. BBB Disruption by Elacridar Was Associated with Induced Activation of NF-κB Pathway in TgSwDI Mouse Brains

**Figure 7:**
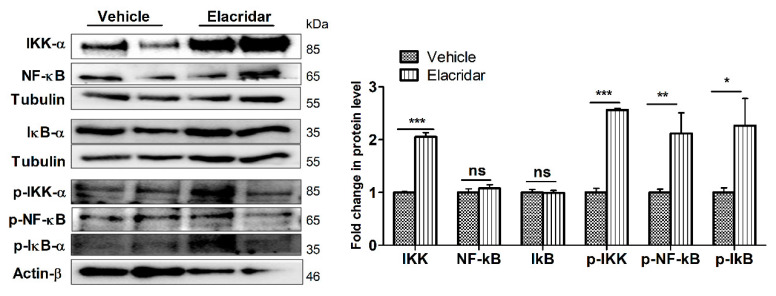
Treatment with elacridar (10 mg/kg) i.p. for 28 days significantly activated the NF-κB pathway in TgSwDI mouse brains. Representative Western blot and densitometry analysis, presented as fold change by elacridar on each protein compared to vehicle treatment, demonstrated that elacridar significantly increased expression of IKK-α, p-IKK-α, p-NF-κB, and p-IκB-α. Statistical analysis was determined by Student’s *t*-test. Data represented as mean ± SEM of *n* = 5 mice per group, ns = not significant, * *p* < 0.05, ** *p* < 0.01, *** *p* < 0.001 compared to vehicle-treated group. kDa indicates the molecular weight of analyzed proteins.

The effect of elacridar on the expression of proteins associated with the NF-κB pathway in mouse brain homogenates was determined by Western blot. As shown in Figure 7, the effect of elacridar was accompanied by a significant increase in the expression of activated NF-κB (p-NF-κB), inhibitor of NF-kB kinase (IKK) complex and its phosphorylated form, and p-IκB proteins.

## 3. Discussion

Age is associated with an increased risk for dementia. Besides, aging is associated with chronic diseases, which necessitate receiving drugs that might interact adversely with each other and/or with endogenous molecules, which could result in irreversible consequences such as cognitive dysfunction and dementia. For example, it has been reported that prolonged use of proton pump inhibitors may increase the incidence of dementia by acting as inhibitors of the enzyme choline-acetyltransferase responsible for the synthesis of the neurotransmitter acetylcholine [28]. Recent findings from our laboratory using a cell-based BBB model to high throughput screen compounds for their ability to enhance the function of the in vitro model [27], we identified several compounds as enhancers of the BBB model function, which protected against Aβ toxicity on the monolayer intactness. These compounds include FDA approved drugs and investigational compounds. Besides enhancers, we were also able to identify disrupter compounds [27]. These results suggest the chronic use of some drugs could alter the barrier function of the BBB, which could potentially affect brain function. To clarify these results further, the objective of this study was to investigate the effect of pharmacological disruption of the BBB on Aβ-related pathology in TgSwDI, a mouse model for CAA and AD. For this purpose, elacridar was used as a model drug that disrupts the BBB function by inhibiting key efflux transport proteins for BBB function and Aβ clearance across the BBB. It has been reported that a single dose of elacridar increases BBB permeability of P-gp substrates via P-gp inhibition [29]. In addition, it has been shown that in an AD mouse model, the administration of elacridar blocks 1α,25 dihydroxyvitamin D3-induced reduction of soluble Aβ brain levels by inhibiting P-gp [30]. However, studies on the effect of BBB disruption by the chronic administration of elacridar, as a model inhibitor compound of P-gp and BCRP, on BBB function and Aβ-related pathology are limited.

Elacridar daily administration for 28 days in TgSwDI mice disrupted the BBB function and increased Aβ brain load with a marked increase in astrocytes activation. Elacridar is an investigational compound in cancer research used to overcome drug resistance and enhance the distribution of chemotherapeutic agents into tumors and organs, including the brain [31]. Elacridar is a highly specific inhibitor of efflux transporters P-gp and BCRP [32]. With aging and in AD, the expression of Aβ major transport proteins including P-gp, LRP1, and BCRP is downregulated, an effect that is associated with reduced Aβ clearance [19,33,34,35,36,37]. This observation suggested that restoring Aβ-BBB transporters could protect the brain from Aβ-mediated pathology [38,39], as supported by preclinical studies reported the upregulation of P-gp and LRP1 induced Aβ clearance across the BBB, an effect that was associated with reduced Aβ and related pathology [40,41,42,43,44,45,46]. On the other hand, compounds that inhibit Aβ transport proteins are expected to reduce Aβ clearance and increase its deposition in mouse brains and microvessels. For example, it has been reported that borneol, a monoterpenoid compound derived from *Dryobalanops aromatica Gaertn f.* and *Blumea balsamifera DC* widely used in traditional Chinese medicine, disrupts the endothelial tight junctions, downregulates P-gp expression, and disrupts the BBB in vitro and in vivo [47]. Similarly, in the current study, P-gp and BCRP downregulation by elacridar treatment disrupted the BBB function and integrity, as demonstrated by the reduced expression of tight junction protein ZO-1 in isolated brain microvessels and the reduced expression of tight junction protein claudin-5 in vitro. These proteins play an important role in maintaining functional BBB and brain homeostasis. Jiao et al. reported that the disruption of claudin-5 alone is enough to cause functional changes in the BBB, which could subsequently allow the inconsistent movement of substances and toxins from the brain to blood and vice versa [48]. In another study, reduced ZO-1 expression was closely associated with the degree of BBB damage and thus was considered as a marker of BBB disruption [49]. In our study, the reduction in tight junction proteins was associated with increased IgG extravasation in mouse brains, indicating a leaky BBB. This in vivo observation was consistent with results from the in vitro data where elacridar significantly reduced LY permeation across the monolayer as well as TEER values.

Several studies reported the role of the BBB in controlling brain Aβ levels [50,51,52,53]. RAGE regulates the influx of peripheral Aβ into the brain [54], while LRP1, P-gp, and BCRP clear brain Aβ from the brain to blood and/or by peripheral extrusion of peripheral Aβ by the efflux transporters [19,33,34,37]. Mice treatment with elacridar reduced expression levels of P-gp and BCRP, but not LRP1, and increased expression of RAGE in brain microvessels, an effect that could contribute to the significant increase in total Aβ and deposits in mouse brains. Furthermore, altered transporters function expressed at the endothelial cells of the BBB increased Aβ deposits on the microvessels as shown by ThioS staining and Aβ immunostaining. Others and we previously reported that P-gp deficiency in mice models of AD suppressed Aβ clearance and increased brain Aβ deposition [42,55]. These findings are consistent with those observed in humans, which demonstrated cerebrovascular expression of P-gp is inversely correlated with Aβ plaque numbers in subjects without dementia [56]. Moreover, BCRP has been shown to play a role in controlling Aβ brain levels by preventing Aβ entry from the blood into the brain across the BBB; in CAA, BCRP expression is upregulated to limit Aβ_40_ access to the brain [57]. Thus, the downregulation of these efflux transporters at the BBB may accelerate parenchymal and vascular Aβ accumulation, which could contribute to AD pathogenesis. In this work, we did not investigate the multidrug resistance-associated protein1 (MRP1), however, there is a potential for elacridar to modulate MRP1 and thus affect BBB function and Aβ brain levels. A previous report showed the complete knockout of *Abcc1*(the gene encodes for MRP1) in an AD mouse model increased Aβ brain deposit, suggesting, like P-gp, the role of MRP1 in controlling Aβ brain levels [58].

RAGE, an immunoglobulin superfamily member, functions as a receptor for a series of ligands, including Aβ [54]. RAGE is known to mediate the entry of circulating Aβ into the brain across the BBB [54]. A significant increase in endothelial RAGE expression was observed in postmortem AD brains compared to controls [59]. Besides downregulating Aβ efflux transporters, elacridar increased RAGE, which could contribute to the increased levels of brain Aβ.

**Figure 8:**
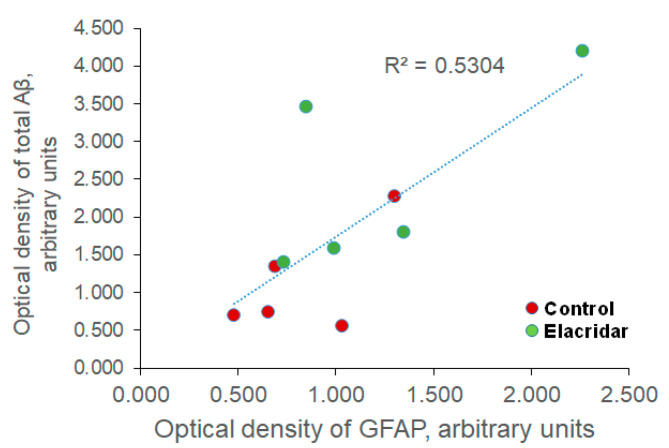
Correlation analysis between optical density changes in GFAP and total Aβ levels revealed a positive correlation between GFAP and total Aβ, as demonstrated by the coefficient of determination (R^2^). *n * =  5 mice/treatment group were used. Optical density is presented in arbitrary units.

In response to inflammation, GFAP intensity increases, and astrocytes remodel into an activated star-like shape [60]. Similarly, our data demonstrated that compared to vehicle-treated mice, the downregulation of P-gp and BCRP by elacridar significantly increased GFAP intensity in mouse brains as determined by immunostaining and Western blotting. While further studies are required, this observed effect could be a direct effect of elacridar on astrocytes activation and/or indirectly through increased brain levels of Aβ as suggested by the correlation analysis between changes in the optical density of GFAP and total Aβ levels, which revealed a positive correlation between GFAP and total Aβ (Figure 8). Thus, based on the in vitro findings showing elacridar to reduce the intactness of the cell-based BBB model, BBB disruption associated with Aβ accumulation could largely contribute to astrocyte activation.

In addition, increased Aβ was associated with increased levels of MMP9, which has been linked to BBB breakdown in neurodegenerative diseases, including AD [61,62]. For example, Yang et al. showed that endothelial cell treatment with Aβ_42_ induced the monolayer permeability by disrupting ZO-1 expression through increased MMP9 secretion in vitro [63], an effect that was also observed in vivo in the brains of 5XFAD mice [63]. Interestingly, however, BBB disruption by elacridar did not alter the expression of synaptic markers suggesting a lag time between BBB disruption and synaptic loss, which could be observed with longer treatment time than 28 days.

To explain the observed effect, we investigated elacridar’s effect on the NF-κB pathway as a potential mechanism for BBB disruption. Data from the in vivo studies showed downregulation of P-gp and BCRP, and upregulation of RAGE accompanied activation of NF-κB pathway in mouse brains. To evaluate elacridar specific effect on the endothelial cells, we performed in vitro studies with bEnd3. Findings showed that cells’ treatment with elacridar reduced P-gp and claudin-5 expression and increased the monolayer permeability as measured by LY assay. These results are consistent with the in vivo findings, which demonstrated increased IgG extravasation. Activation of inflammatory NF-κB signaling pathway has been shown to compromise the BBB-endothelium function by reducing P-gp and increasing RAGE expressions [48,64,65], and activate inflammatory mediators that facilitate disease progression [66,67].

Collectively, these findings demonstrated the pharmacological disruption of BBB could lead to CAA and AD, and possibly other vascular disorders such as cerebral small vessel disease (CSVD) [68].

However, while our studies demonstrated the pharmacological disruption of BBB by elacridar, used as a model compound, on enhancing Aβ and related pathology in TgSwDI male mice, additional experiments in wild type mice as well as in female TgSwDI mice would be important to perform to understand elacridar treatment effect in the absence of pathology and whether this effect is sex-dependent.

## 4. Materials and Methods

### 4.1. Materials and Chemicals

Elacridar and dithiothreitol (DTT) were purchased from Sigma-Aldrich (St. Louis, MO, USA). Dulbecco’s modified Eagle’s medium (DMEM), sterile phosphate-buffered saline (PBS), and penicillin/streptomycin antibiotics were obtained from Gibco (Grand Island, NY, USA). Fetal bovine serum (FBS) was purchased from Atlanta Biologicals (Flowery Branch, GA, USA). Total protein measurement reagents using the bicinchoninic acid (BCA) method were obtained from Pierce (Rockford, IL, USA). Antibodies used were mouse monoclonal antibody against light chain LRP1 (Abcam, Cambridge, MA, USA), mouse monoclonal antibody C-219 against P-gp from BioLegend (San Diego, CA, USA), mouse monoclonal antibody against BCRP (Cell signaling; Boston, MA, USA). Monoclonal antibodies for claudin-5, ZO-1, and HRP-labeled secondary antibodies were purchased from Invitrogen (Carlsbad, CA, USA), goat polyclonal antibodies against actin (C-11) and Matrix Metallopeptidase 9 (MMP9) were purchased from Santa Cruz Biotechnology (Dallas, TX, USA). Antibodies for pre- and postsynaptic proteins SNAP-25 and PSD-95, respectively, were purchased from GeneTex (Irvine, CA, USA), antibodies for I*κ*B-α, p-I*κ*B-α, NF-кB p65, and p-NF-кB p65 were purchased from Cell Signaling. All other reagents and supplies were purchased from VWR (West Chester, PA, USA) and Fisher Scientific (Hampton, NH, USA).

### 4.2. Cell Culture

The immortalized mouse brain endothelial cell line, bEnd3, was obtained from ATCC (Manassas, VA, USA). bEnd3 cells, passage 25–35, were cultured in DMEM supplemented with 10% FBS, penicillin G (100 IU/mL), streptomycin (100 g/mL), 1% *w*/*v* nonessential amino acids, and glutamine 2 mM. Cultures were maintained in a humidified atmosphere (5% CO_2_/95% air) at 37 °C, and media were changed every other day.

### 4.3. In Vitro Permeability Assay

The permeability assay across bEnd3 cells monolayer was performed as we reported previously [27,69]. In brief, cells were seeded on inserts of HTS transwell 96-well plate (Corning, NY, USA), coated with 50 μL of fibronectin solution (30 μg/mL in PBS) as a basement membrane substitute. bEnd3 cells were seeded at a density of 50,000 cells/cm^2^ on the apical side, and 200 μL of fresh media were added to the basolateral side. To achieve optimal barrier integrity of bEnd3 cells, cells were incubated at 37 °C, 5% CO_2_ for 5 days [27]. The effect of elacridar on the barrier integrity of bEnd3 cells was evaluated on the sixth day of seeding by monitoring TEER measurements and by LY assay as described below.

Cells were treated with different concentrations of elacridar (dissolved in DMSO and maintained at 0.1% in all in vitro experiments) starting on day three and four of cell seeding, which correspond to treatment time of 48 and 72 h, respectively. The evaluated concentrations were in the range of 1–10 µM. Vehicle treated cells (as control) were treated with 0.1% DMSO. At the end of the treatment period (day 6 post-seeding), the integrity of the endothelial barrier was evaluated by measuring monolayer tightness through TEER, and permeability of LY across the monolayer. For LY permeability, 50 μL of LY (100 μM) diluted in transport buffer (141 mM NaCl, 4 mM KCl, 2.8 mM CaCl_2_, 1 mM MgSO_4_, 10 mM HEPES, and 10 mM d-glucose, pH 7.4) were added to the apical side, while the media in the basal side (lower chamber) were replaced with 200 μL of warmed transport buffer. One hour later, the concentrations of LY in the apical and basal sides were determined by measuring LY fluorescence intensity at excitation and emission wavelengths of 485 and 528 nm, respectively, by using a Cytation-5 microplate reader (Biotek, VT, USA) supported with Gene5 software (Biotek) for data acquisition. To calculate the apparent permeation coefficient (Pc) of LY, the following was used:

Pc (cm/sec) = (V_b_ × C_b_) / (C_a_ × A × T) (1)

where, V_b_ is the volume of the basal side (200 μL), C_b_ is the concentration of LY (μM) in the basal side, C_a_ is the concentration of LY (μM) in the apical side, A is the membrane area (0.143 cm^2^), and T is the time of transport (3600 s). LY concentration was calculated against a standard curve prepared by measuring the fluorescence intensity of various concentrations of LY [27]. While for TEER measurements, an automated TEER measurement system with Corning HTS Transwell-96 (REMS-96C) electrodes (World Precision Instrument; Sarasota, FL, USA) was used. To reflect the actual readings of the bEnd3 cell layers, the TEER of blank inserts was subtracted from the measured TEER of each experimental insert. Values were expressed as Ω·cm^2^.

### 4.4. Preparation of Cytosolic or Nuclear Extracts from bEnd3 Cells

Cytosolic and nuclear extracts were obtained as previously described [70]. bEnd3 cells were plated at a density of 1 × 10^6^ cells in 100 mm dishes and cultured for 24 h; subsequently, the cultures were treated with or without 5 µM elacridar for an additional 4 and 24 h. After each time point, cells were scraped off the plates, collected by centrifugation, and washed with PBS. The cell pellet was resuspended in cytosol extraction buffer consisting of 10 mM HEPES (pH 7.5), 10 mM KCl, 0.1 mM EDTA, 1 mM DTT, 0.5% Nonidet-40, and 0.5 mM phenylmethylsulfonyl fluoride (PMSF) protease inhibitor, and allowed to swell on ice for 20 min with intermittent mixing [70]. Tubes were vortexed to disrupt the cell membrane, and the homogenate was centrifuged at 12,000× *g* for 10 min at 4 °C. The supernatant (cytosolic extract) was collected and frozen at −80 °C until use. The pellet was further treated for nuclear protein extractions. The nucleus pellet was resuspended in an equal volume of nuclear extraction buffer consists of 20 mM HEPES (pH 7.5), 400 mM NaCl, 1 mM EDTA, 1 mM DTT, and 1 mM PMSF with protease inhibitor. The lysing nucleus was left on ice for 30 min and then centrifuged at 12,000× *g* for 15 min at 4 °C. The supernatant (nuclear extract) was removed and stored −80 °C until analysis.

### 4.5. Animals Treatment

All animal experiments and procedures were approved by the Institutional Animal Care and Use Committee of the University of Louisiana at Monroe and according to the National Institutes of Health guide for the care and use of laboratory animals (NIH Publications No. 8023, revised 1978). Surgical and treatment procedures were consistent with the IACUC policies and procedures. Male TgSwDI mice (Jackson laboratory; Bar Harbor, ME, USA) aged 4 months were used. All mice were housed in plastic containers under the conditions of 12 h light/dark cycle, 22 °C, 35% relative humidity, and *ad libitum* access to water and food. This mouse model expresses human APP KM670/671NL (Swedish), APP E693Q (Dutch), APP D694N (Iowa) mutations. TgSwDI mice develop fibrillary amyloid deposits primarily in the cerebral microvasculature starting at age 2–3 months and extensively at 12 months of age [39]. Aβ deposition also occurs in the brain parenchyma of these mice, generally in the form of diffuse plaque-like structures beginning at approximately 3 months of age in the subiculum, hippocampus, and cortex, as well as gliosis associated with an increase in GFAP-positive astrocytes and activated microglia that progress with age [39]. In addition, the early accumulation of Aβ on TgSwDI mouse brain microvessels at 3 months of age correlated with early memory deficits [71]. TgSwDI mice also exhibit a profound age-dependent BBB dysfunction, starting at 3 months of age, due to vascular deposition of Aβ causing an impaired regulation of the brain circulation [72]. Thus, in this study, TgSwDI mice were used at the age of 4 months, where TgSwDI mice already exhibit BBB disruption, Aβ vascular deposition, and Aβ related pathology.

Mice were divided into two groups (*n* = 5 mice per group), a vehicle-treated group received an intraperitoneal injection (i.p. injection of 100 µL) of the vehicle used to dissolve elacridar composed of 70% water: 16.5% DMSO: 13.5% PEG 400, and the treatment group received elacridar (10 mg/kg/day, i.p. injection of 100 µL) for 28 days. Elacridar dose and route of administration were selected based on previous reports [30]. Elacridar is a third-generation dual P-gp and BCRP inhibitor [32]. During the treatment period, animals’ body weights were measured weekly; health status and normal behavior were checked daily. Mice body weights were not significantly different between the treatment and control groups and were in the range of 28.5 ± 2.5 to 26 ± 2.4 g, respectively. At the end of treatment, mice were intraperitoneally anesthetized with ketamine and xylazine (125 and 20 mg/kg, respectively), followed by decapitation to collect brain tissues.

### 4.6. Isolation of Brain Microvessels

Brain microvessels were isolated as described previously [69]. Brain hemispheres were homogenized in ice-cold DPBS followed by the addition of one volume of 30% Ficoll 400 (Sigma-Aldrich). Homogenate was centrifuged at 8000× *g* for 10 min, and the resulting pellet was suspended in ice-cold DPBS containing 1% BSA and passed over a glass bead column to collect microvessels adhering to the glass beads. Isolated microvessels were used to determine the expression of tight junction proteins ZO-1 and claudin-5, and Aβ transport proteins P-gp, BCRP, RAGE, and LRP1 by Western blot.

### 4.7. Western Blot for Cell Lysate, Brain Homogenate, and Microvessels

The total protein for each sample was determined using the BCA protein assay. Protein samples (25 μg) were loaded and resolved on 10% SDS-polyacrylamide gel, then transferred electrophoretically onto PVDF membranes. Membranes were incubated in 1% blocking solution followed by overnight incubation at 4 °C with primary antibodies. Analyzed proteins for cytoplasmic and nuclear fraction lysates from in vitro experiments included IкB-α (1:1000 dilution), NF-кB (p65; 1:1000 dilution), phosphorylated IкB-α (1:1000 dilution), phosphorylated NF-кB (1:1000 dilution), and housekeeping proteins tubulin (cytoplasmic) and histone H3 (nuclear) (1:500 dilution). For brain homogenate and isolated microvessels samples, proteins analyzed were P-gp (1:200 dilution), BCRP (1:200 dilution), LRP1 (1:5000 dilution), synaptic markers (PSD-95 and SNAP-25), RAGE, MMP9, GFAP (1:500 dilution), tight junctions (ZO-1 and claudin-5), tubulin and actin-β from Invitrogen. For detection, the membranes were washed free of primary antibodies and incubated with HRP-labeled secondary IgG anti-mouse antibody for P-gp, PSD-95, RAGE, GFAP, MMP9, and claudin-5; anti-rabbit antibody for LRP1, ZO-1, BCRP, and SNAP-25. Similar antibodies to those used to probe NF-кB pathway proteins in cell lysates were also used for homogenate lysates. All secondary antibodies were from Invitrogen.

Protein blots were developed using a chemiluminescence detection kit (Thermo Fisher Scientific). Bands were visualized using ChemiDoc imaging system (Bio Rad; Hercules, CA, USA) and analyzed by Image Lab software v 6.0 (Bio-Rad). The results were expressed as the fold change in protein level compared to the control group after normalization to the housekeeping proteins.

### 4.8. Immunohistochemical Analysis

All cryostat brain slices (16 μm) were methanol-fixed then blocked for 30 min with 10% normal donkey serum in PBS. Double immunostaining of microvessels with Aβ was performed using rabbit polyclonal collagen-IV antibody (Millipore; Temecula, CA, USA) at 1:200 dilution detection followed by IgG-CFL 594 conjugated donkey anti-rabbit (Santa Cruz Biotechnology) as a secondary antibody. For Aβ detection, Alexa fluor-488 conjugated anti-Aβ antibody (6E10; BioLegend) at 1:200 dilution was used.

To assess IgG extravasation from brain microvessels, brain sections were fixed and blocked, as described above, then probed by dual immunohistochemical staining for collagen-IV and mouse IgG using rabbit anti-collagen-IV, and fluorescein-conjugated donkey anti-IgG to detect IgG (Santa Cruz Biotechnology), both at 1:200 dilution. To evaluate astrogliosis, brain sections were fixed and prepared as above. Double immunostaining was performed using GFAP antibody for astrocytes at 1:100 dilution and Alexa fluor-488 labeled 6E10 for total Aβ at 1:200 dilution.

Images were captured and adjusted to the lowest background signal using Nikon Eclipse Ti-S inverted fluorescence microscope (Melville, NY, USA). For quantification of total Aβ load, IgG extravasation, and GFAP, sections were normalized to the same background for hippocampi and cortexes regions. Images were analyzed by Image J software version 1.44 (National Institutes of Health, Bethesda, MD, USA) that was set for mean value, minimum value, maximum value, and limit to the threshold followed by analysis. Results showed the mean value, minimum value, maximum value, and area. The minimum value was similar in all sections; the mean value (presented as density) was then plotted as the fold change caused by elacridar compared to the vehicle-treated group.

### 4.9. Statistical Analysis

All values were expressed as mean ± SEM for in vivo data and as mean ± SD for in vitro data. Statistical analysis was done with Prism v5.0 software (Graphpad). The statistical significance for all results was assessed by one-way ANOVA for multiple groups and Student *t*-test for two groups compared to the control group. A *p*-value of <0.05 was considered statistically significant.

## 5. Conclusions

In conclusion, findings from this study suggest the pharmacological downregulation of BBB-suited P-gp and BCRP could disrupt the BBB function and increase Aβ brain accumulation and thus have the potential to increase the risk of AD and CAA. BBB disruption by elacridar is mediated by activation of the NF-κB pathway, which induces BBB permeability by increasing MMP9 and thus downregulating the expression of tight junction protein ZO-1.

## Data Availability

The data presented in this study are available within the article text and figures.

## Abbreviations

Aβ	amyloid-β
AD	Alzheimer’s disease
APP	amyloid precursor protein
BBB	blood brain barrier
BCA	bicinchoninic acid
BCRP	breast cancer resistant protein
CAA	cerebral amyloid angiopathy
CSVD	cerebral small vessel disease
DTT	dithiothreitol
GFAP	glial fibrillary acidic protein
HTS	high throughput screening
IκB	inhibitor of NF-κB
IKK	inhibitor of NF-κB kinase
LRP-1	low-density lipoprotein receptor related protein-1
LY	Lucifer yellow
MMP9	matrix metallopeptidase 9
P-gp	P-glycoprotein
PSD-95	postsynaptic density protein-95
RAGE	advanced glycated end products
SNAP-25	synaptosomal-associated protein-25
TEER	transendothelial electrical resistance
